# Fathers’ involvement in perinatal healthcare in Australia: experiences and reflections of Ethiopian-Australian men and women

**DOI:** 10.1186/s12913-021-07058-z

**Published:** 2021-09-30

**Authors:** Faye Forbes, Karen Wynter, Berihun M. Zeleke, Jane Fisher

**Affiliations:** 1 Global and Women’s Health, School of Public Health and Preventive Medicine, Monash University, 553 St Kilda Rd, Melbourne, Victoria 3004 Australia; 2 Faculty of Health, School of Nursing & Midwifery – Western Health Partnership, Deakin University, Melbourne, Victoria 3000 Australia; 3 School of Public Health and Preventive Medicine, Monash University, 553 St Kilda Rd, Melbourne, Victoria 3004 Australia; 4 College of Medicine and Health Sciences, Institute of Public Health, University of Gondar, Gondar, Ethiopia

**Keywords:** Father inclusive, Male partner involvement, Perinatal healthcare, Culturally and linguistically diverse, Maternity care, Migrant, Pregnancy, Childbirth, Father involvement, Qualitative

## Abstract

**Background:**

Family-centred maternity care models include the expectation that fathers prepare for and attend the birth. In Australia over 20% of the population is from a culturally and linguistically diverse background. Public policies espouse culturally competent healthcare. Little is known about the experiences of perinatal health care of men from culturally and linguistically diverse (CALD) communities living in high income countries. The aim was to understand the experiences, attitudes and beliefs about father’s inclusion in perinatal healthcare, from the growing, and recently settled community of Ethiopian families living in Australia.

**Methods:**

A qualitative study using semi-structured individual interviews with Ethiopian-Australian men and women who had experienced Australian maternity care and were sampled for diversity of time since migration, and parity. Interviews were in English, audio-recorded, transcribed and then analysed thematically.

**Results:**

Participants were seven women and six men all born in Ethiopia, including two couples. Key themes included: the loss of extended family through migration, new roles for both parents and the need to establish ‘family-like’ relationships with friendship groups in Australia. There was a willingness to involve male partners in the Ethiopian community in Australia, although it was recognised as a cultural change. Experiences of male partner involvement were mixed among healthcare types, with men attending Maternal and Child Health (MCH) appointments less frequently than antenatal (ANC) appointments.

**Conclusions:**

Results suggests men may be missing out on the education provided during antenatal appointments and may benefit from an alternative. There were not universally high levels of cultural competency among healthcare professionals, with further training still required. Commitment to paid employment remains a barrier to men’s involvement, suggesting that flexible working conditions and increased paternity leave would support their involvement. Alternatively services could utilise flexible delivery methods such as phone and zoom to include fathers.

## Background

Perinatal health services are encouraged to deliver care that is inclusive of fathers [1, 2]. The inclusion of fathers is underpinned by evidence of the positive impact of father involvement on women’s mental health, child development and the opportunity to engage men in their own healthcare needs. A woman’s relationship with her intimate partner is a prominent determinant of her perinatal mental health [3]. Positive involvement from the father (respectfully engaged, accessible and responsible behaviours) is associated with good child outcomes in several parameters such as rates of pre-term birth and low birth weight [4], eating behaviours, language acquisition and vocabulary [5], psychological problems, cognitive development and externalising behaviour problems [6]. Involving fathers during their partners’ pregnancy, childbirth and the child’s early infancy has been suggested as a method of supporting the father-child relationship from the earliest possible stage [7, 8]. It also offers an opportunity to provide education or resources regarding their mental health.

Family-centred maternity care models include the expectation that fathers prepare for and attend the birth (such as antenatal education classes and present during the birth) [9]. Perinatal clinical staff report that many agree their role includes engaging men [10, 11]. Yet fathers are not always actively included in perinatal healthcare appointments [12]. A review of Swedish research, reported men feeling unsupported through the perinatal period [13]. A review of parent education programs in the USA showed that few programs included fathers [14]. A meta-synthesis of qualitative research of father’s experiences in maternity care in high resource countries described a discord among fathers views of themselves as ‘parent and partner’ and the way they felt in maternity care ‘not a patient, not a visitor’ [15].

Australia is a diverse, multicultural country. Approximately 28% of Australian permanent residents are born outside of Australia and 21% are from a culturally and linguistically diverse (CALD) background [16]. This proportion is projected to increase as population growth due to migration overtakes growth from births within the country, and an increasing proportion of visas are allocated to individuals from non-English speaking countries [17]. The Ethiopian community is a growing cultural group within Australia, hosting over 11,000 members of which over 50% reside in Melbourne. The community contains a diverse mix of people arriving via refugee and skilled migration schemes, with the majority of people arriving in the last 10 years [16].

As a multicultural country, the Australian government aims to provide culturally competent healthcare services [18]. The most commonly used definition for cultural competency states “cultural competence is a set of congruent behaviours, attitudes, and policies that come together in a system, agency, or amongst professionals and enables that system, agency, or those professionals to work effectively in cross-cultural situations” [19]. Specific strategies or programs designed to support engagement among culturally diverse communities include making interpreters available if required during healthcare appointments, and employer-led cultural competency training.

In order to assess the cultural competency of healthcare delivery to fathers, it is essential to conduct research with culturally and linguistically diverse communities. There are a number of indications CALD families have particular healthcare needs. Gender roles within families are strongly influenced by culture. An emphasis on father involvement during the perinatal period (as is now common in high income Western countries) is a relatively new phenomenon for many cultural groups [20, 21]. Evidence suggests that culturally diverse groups such as refugees are at greater risk for psychological distress during the perinatal period [22], access less care and tend to have more negative experiences [23].

However, much of the research into father involvement in the perinatal period in high income settings has been conducted in middle class English-speaking communities [24]. There are few investigations of father involvement in the perinatal period among culturally and linguistically diverse (CALD) communities in high income countries.

Moreover there are very few studies investigating cultural communities individually. The term “culturally and linguistically diverse” includes a tremendous diversity of cultures, social norms and experiences. In order to better understand whether and in what circumstances to group cultures together, an exploration of individual cultures is required [25].

A body of research has been conducted in low and middle income settings, which describes some of the benefits and harms from including male partners in perinatal healthcare, as well as the barriers and enablers. A review of barriers to male partners’ involvement in maternal healthcare described the main determinants of involvement as: sociodemographic factors such as education and income; health service factors such as opening hours, behaviour of health professionals in accommodating men; and cultural factors such as communication between men and women, beliefs and attitudes [26]. The review describes cultural attitudes specifying that pregnancy, childbirth and early infancy is considered in many cultures in Sub-Sahara Africa to be a woman’s domain.

Our current understanding of culturally diverse father’s role in perinatal health services in Australia is captured in women’s experiences and how they refer to their partner’s involvement. The evidence comes from qualitative and quantitative studies in high income settings with women from the Middle East, South and Central Asia and Africa. In some qualitative research the role of the father emerges as a key theme [27, 28], in others it was mentioned only briefly [29, 30], while in some studies (particularly structured quantitative research) the role of the father was not mentioned at all [31, 32].

The most common experience reported by women about immigrant fathers was that the fathers’ role in relation to pregnancy, childbirth and the postnatal period had changed in comparison to in their home country [28, 33]. Several qualitative studies included accounts from women stating that it would have been unusual for their partner to be present at the birth in their country of origin, but they enjoyed having them at the birth in Australia [27, 34, 35]. There were some accounts of their husbands not feeling welcome [36] or feeling judged. For example, women who had experienced female genital mutilation felt themselves or their husbands might be judged in hospital in their new countries [37].

There was a general consensus that women experienced an increased reliance on their partner for support during the perinatal period after migration [38, 39]. Qualitative research suggests this was primarily because they lacked support from extended family and community that they would have experienced in their home country [27, 28]. A survey of close to 10,000 women indicated the partner was the most common source of support to both non-English speaking women and English speaking women in the perinatal period [27]. On the other hand, another common theme among the studies was the pressure men experience to earn money. In numerous qualitative studies, women mentioned that their partners needed to work long hours and were thus unable to provide enough support to ‘fill the gap’ left by absent extended family or ‘female kin’ [27, 35, 40]. This was supported by statements in other research that indicated that many men still considered themselves as providers for the family rather than nurturers [34, 41].

Focus groups and interviews held with women from a CALD background made reference to women’s reliance on their male partner to translate information at perinatal appointments or provide knowledge of services [42]. The authors referred to health professionals not arranging for interpreters. Translators were often not used by health professionals and instead nurses, midwives and other staff relied on men to translate important information for their partners [28, 33, 40, 43].

Lastly research identified that significant proportions of women from a CALD background in the communities researched were divorced or widowed and did not have a male partner [36, 44, 45]. In these cases, suggesting strategies to support CALD families through father involvement in perinatal services would not be appropriate, and special care should be taken not to alienate single women, as they may already experience the absence of a male partner as a barrier to accessing care [37, 44].

In summary, there are descriptions of experiences reported by women from CALD backgrounds in relation to the perinatal period and perinatal health services. However, the evidence about the outcomes, experiences and attitudes of *men* from such communities in relation to *their involvement* in perinatal health services is extremely sparse.

Furthermore the contribution of evidence regarding specific cultural groups (as opposed to generalisations among all non-dominant cultural groups) is limited. There are no studies exploring the experiences of Ethiopian-Australian fathers whose needs and perspectives are therefore poorly understood.

The aim was to describe the experiences, attitudes and beliefs of men and women originally from Ethiopia living in Australia, in relation to father involvement with perinatal health services during pregnancy, childbirth and the postnatal period.

## Methods

### Approach

Qualitative research was considered an appropriate approach given the emerging status of knowledge into culturally diverse father’s experiences in pregnancy, childbirth and infancy care in Australia. The authors decided that focusing on one cultural group and sampling for diversity within that group would allow a deeper exploration of these father’s experiences.

### Design

An exploratory investigation using a qualitative method (semi-structured interviews).

### Setting

Australia has a two-tiered health system: all citizens are entitled to fee free hospital care in the public health system and people can purchase private health insurance that entitles them to care provided by a clinician of choice in a private hospital. Several models of public maternity care are offered: 10 standard antenatal care consultations with public hospital midwives, General Practitioners or if needed, hospital-based obstetrician). Births are in hospital, attended by obstetricians and midwives with follow), nurses and midwives during childbirth. Nearly half of all women receive private sector care provided by private obstetricians in private hospitals.

In Victoria, the Australian state in which this study was conducted, postnatal care is provided in a universal system by community-based Maternal and Child Health (MCH) nurses tracking the health and development of their child at 10 key ages and stages [46]. Men are able to access 10 days paid government paternity leave if they have been in paid employment for 10 months prior to the birth.

The community Maternal and Child Health (MCH) services program aims to be “father inclusive”. The Maternal and Child Health program standards recognise “the unique role of the father in the health and development of the child and supports him in his role” (pp 21) as a key criterion in achieving standard 2 “optimal health and development” [46].

### Definitions

Perinatal healthcare is defined as healthcare provided during pregnancy, childbirth, post-partum and care for an infant in the first year. Fathers’ involvement is defined as a father’s experience attending or attitudes towards, perinatal healthcare.

### Inclusion criteria

Men and women born in Ethiopia, permanently living in Australia with sufficient English fluency to read participant information and consent form and participate in an interview, who had experienced or their partner had experienced pregnancy and childbirth in Australia in the last 2 years were eligible to participate.

### Recruitment

Participants were recruited via a snowball sampling technique [47]. Community leaders from within the Ethiopian community identified two potential participants and provided an initial introduction to the researcher (by setting up a face-to-face meeting or providing telephone details with the permission of the potential participant). On completion of the study, participants were asked if they are able to identify other potential participants and provide an introduction in the same way. One of the first participants lived in Australia following a skilled migration pathway, where he came on a student visa. Another early participant arrived in Australia on a refugee visa. This led to recruitment from different parts of the community, parity and duration of time in Australia, which allowed the researchers to recruit for diversity. Recruitment continued until the data was deemed to have sufficient “information power” [48]. The concept of “information power” is commonly used in qualitative research to help determine the sample size required. Sufficient information power is thought to have occurred during data collection, when no new themes emerge from subsequent interviews [48].

### Interview procedure

Semi-structured interviews were held in person or using an online platform such as Zoom by the first author. Interviews were conducted in English. Audio-recorded oral informed consent was received for the interview process and audio recording. All participants (including couples) were interviewed individually. They were assured their stories would be kept confidential and no one outside the research team would have access to their transcript.

### Data source

An interview script was used as a guide for the conversation, containing open-ended questions about male partners’ involvement and experiences during pregnancy, childbirth and the infant’s first year. For example (for women’s partners): *“Please describe what your partner’s pregnancy for your youngest child was like for you”, “Tell me about your experiences of your partner’s pregnancy care, e.g. antenatal clinic?”, “What were your reasons for being involved?”* and *“Overall, please tell me what you think about men’s participation in family health services?”*

### Data transcription and analysis

Audio data were de-identified and transcribed using a combination of an automated method and manual transcription. Thematic analysis was conducted using a line-by-line deductive coding process, facilitated by Nvivo software. Codes were then organised into similar categories that became the themes. Men’s and women’s data were analysed separately, and then compared and merged as described by Braun and Clarke [49].

### Reflexivity

The researchers underwent a reflexive process to identify potential biases in order to ‘bracket’ these and remain objective [50]. The first author, who conducted interviews and performed the bulk of the analysis, identified beliefs about the benefits and positive nature of male partner involvement in the perinatal period. In order to actively seek alternative viewpoints, questions about potential negative impacts of male involvement were introduced during the reflective process during the interview. During analysis themes that may reflect negative consequences of male partner involvement were actively sought.

## Results

Participants included seven women and six men all born in Ethiopia, including two couples. Participant’s demographic information is displayed in Table 1.

**Table 1:** Participants demographic information

ID	Gender	Relationship status	Original visa type	Number of children	Years in Australia
M1	Male^a^	Married cohabitating	Student/ Skilled	1	5
M2	Male	Married cohabitating	Student/ Skilled	2	5
M3	Male^b^	Married cohabitating	Refugee	3	10
M4	Male	Married cohabitating	Student/ Skilled	2	5
M5	Male	Married cohabitating	Student/ Skilled	2	8
M6	Male	Married cohabitating	Refugee	5	10
F1	Female^a^	Married cohabitating	Partner/ Skilled	2	5
F2	Female^b^	Married cohabitating	Partner/ Refugee	3	7
F3	Female	Married cohabitating	Refugee	3	7
F4	Female	Married cohabitating	Refugee	3	15
F5	Female	Separated by work	Refugee	2	8
F6	Female	Separated permanently	Partner	3	7
F7	Female	Married cohabitating	Refugee	3	14

^a,b^Man and woman in a relationship

### Summary of themes and sub-themes

#### New experiences following migration: comparison between Ethiopia and Australia and the social context around birth

Men and women both made comparisons between the cultural norms around pregnancy and childbirth in Ethiopia and in Australia; most had directly experienced previous pregnancies and birth overseas. In general, participants explained that healthcare facilities in Ethiopia were more crowded and staff were often less respectful of families in their care than Australia

*“I know like there are experiences in Ethiopia where the nurses, the doctors are really…really unfriendly. So in comparison, this is really, really good …” Man M2*

Both men and women clearly articulated that fathers were more involved with their children in Australia. Men described having a much closer bond and knowing details of their children’s lives they would not have known previously in their home country. When asked about their attendance at perinatal healthcare appointments both men and women said the male partner had participated more in the Australian setting than they would have had they stayed in Ethiopia.

*“But here, you go inside the maternity room and you see when the baby comes out. That's not in back home, they'd be coming out only the midwives would go with them….. [Here] they allow me to cut the umbilical cord and cut with the scissors. Yep It’s good and it's a bit painful to see all that drama.” Man M3*

One of the key reasons provided for greater levels of male partner involvement in perinatal healthcare in Australia was the changes in social networks around the couple. Almost all participants described the role of extended family and community in Ethiopia in relation to supporting the family during this time. Many went on to explain that the absence of extended family in Australia left them feeling isolated and lacking in support.

*“In terms of cultures even the two countries are very different. You need to know when they're [the birth parents] ready to have a child. It is more of your mother’s and father’s [the grandparents] responsibility than.. your responsibility. .. But here ...we don't have any extra extended family. So you are responsible for everything, for everything.” Man M4*

Many felt that without this network, pregnancy, childbirth and infancy could be demanding for parents in Australia.

*“Compared to back in home [in Ethiopia].. there would be so many people helping. Yeah. So many people to give us support. So [here] I have to be next to her, to give her support.” Man M2*

*“I mean in Ethiopia it is totally on the mother to bring the child and father is the breadwinner mostly. So he wouldn't be around during the day.” Man M2*

In Australia, it was common that both men and women formed strong networks of friends among the Ethiopian community. Several people said that they had formed a network of friends that felt like family.

*“It's almost funny because when you don't have anyone around you, you stick each other. You have to pick someone really cut-out like a family, and be like family” Woman F6*

*“Even though we are friends, but we read like we are family members.… We are very close to each other. Yeah. We support each other like at times like these.” Man M4*

However, some people had their children soon after arriving and before they had a chance to form solid networks and they felt particularly isolated.

*“Yeah. When we are coming here you know, I'm pregnant. After six months I have the baby. I didn't know anyone. I miss my family. I don't know the Australia system. Many things. That time too hard for me. I’ve got my husband to help me, but still I’m feeling like too hard.” Woman F3*

#### Mixed experiences of perinatal healthcare

Many of the families described pregnancies that were planned, with both members of the couple happy to welcome a child.

Some women had been pregnant and gave birth very soon after arriving in Australia, where their living situations and social networks were unstable and they were separated from their partners through the migration process.

*“My first son, my husband wasn't here. He was overseas. That time was really hard time for me even I can remember it was my social worker was with me. I was homeless at that time. I was in the traditional house, like emergency house. Until they gave me a house, I was in emergency house and then three houses at the same time out at that time.” Woman F5*

There was variation in male partner involvement in perinatal healthcare among the stages of pregnancy, childbirth and infancy. It was quite common for male partners to attend antenatal care in Australia. The reasons given were frequently related to emotional support, logistical support like translating English language or driving and to support their wife because she felt unwell. The experiences were mixed, some positive and some negative:

*“Frankly speaking, I don't think that there was a problem [discrimination] because we are Ethiopians. I don't see such a problem, I guess. In many of our visits, we were welcomed. They were open. We can ask whatever questions we do have.” Man M4*

A male participant commented that the doctors conducting antenatal care often seemed rushed and focused on purely medical issues, while the midwives were much kinder and easier to relate to.

*“But when we visit the midwife they have a lot of time to address all the questions that we do have. So frankly speaking, I would love to chat with a midwife instead of the doctors.” Man M4*

With the exception of the two female participants who were separated from their partners, all other participants said the male partner had attended the birth. Overall, the births were described in positive terms, although both men and women recognised that witnessing the birth was very stressful for male partners as well as painful and taxing for women.

*“For me, it's really stressful. One of the very stressful moments in my life, because for most of the birth, I've been in there for the whole time. And it's really painful to watch. And it is stressful. Yeah, both of the births like for me personally is like a very stressful experience for her.” Man M2*

Participants reported a range of hospitals and birth types but both men and women praised the facilities and the midwives assisting at the births of their babies. Male partners described being accommodated during birth; one man had the couple’s two-year-old daughter at the birth and described nursing staff finding her a mattress to sleep on. Another said he was invited to cut the umbilical cord, while another described being kept informed as rapid decisions were made about an emergency caesarean birth. Participants used emotive terms like ‘wonderful’ and ‘awesome’.

*“It's really good. Like the health system and the support from the nurses and from everyone involved in the hospital. Really magnificent. I would say.” Man M2*

*“So this is a really nice place. So I mean I can't ask for any better service. I remember they are really very caring and understand our needs. And they know like everyone is different and they (support) what is best for any expressions of ourselves.” Man M1*

In general, most male partners were not able to attend many, if any, Maternal and Child Health appointments during the baby’s first year. Many men said they would have liked to attend but were not able to because of income-generating work commitments. Many women said that they were happy for their partners to go with them, but usually did not see it as important enough to miss work to attend. Appointments during the baby’s first year seemed less accessible to men, compared to antenatal appointments.

During the COVID-19 lockdown, one man was excluded from the post-natal care appointments when the nurse explained she would only meet with the mother.

*“I haven’t for most of them. He [the baby] mostly just went with mom.” Man M2*

#### Varied experiences of cultural competency among healthcare staff

There were mixed reports of cultural competency among healthcare professionals. Men and women described experiences of interactions where healthcare professionals made participants feel culturally understood, accepted and comfortable. For example, one male partner described a midwife calling him, at the request of his partner, to discuss her condition following a miscarriage.

*“She's good because she told me the truth [about the need for an induced abortion]. … Because she asked me in terms of my religion, is that allowed or not? Oh, I know it's not allowed. But instead of suffering too much after that for us yeah, I took a risk.” Man M3*

Another example was from a female participant with her first child. She had been in Australia with her husband for only a short time and did not have extensive social networks or a developed knowledge of Australian culture, language and services. The Maternal and Child Health nurse assigned to her was able to make her feel comfortable, convey information and in her own words gave her confidence to attend the first-time mothers’ group the nurse organised.

*“And then she tell me everything. She gave me more confidence. And then she sent me to the mother’s group that she organizing….She helped me a lot.” Woman F1*

However, there were also experiences of poor cultural competency among the healthcare professionals. A male participant described the challenges he experienced in accessing antenatal care as an international student. He had a particularly poor experience when his wife was receiving care for pregnancy complications at a private clinic.

*“I just straightforward I fainted [at ANC] because of the way he communicated. It was such stressful information and he was just simply talking about OK, this is just going to be such and such problems. And all he [the doctor] thinks about, I remember, was whether you can afford it. And I felt insulted like it’s not about affording or things like that. What's at stake is the life of my wife the life of my child. I don't think about that I didn't want him to get me just by the look of my face. I mean I don't think he would have done that for an Australian. I come in [after] having a look at me, he just simply said OK I'm not quite sure if you can afford that.” Man M1*

#### The roles and responsibilities for male partners in perinatal healthcare

There were certain roles that male partners performed as a part of their involvement with perinatal healthcare. A very common description of why they were involved was because they wanted to offer emotional support to their partner, and they felt going together supported the relationship.

*“Because during pregnancy, there is a lot of feeling, so most of the time I need to be together with her because we don't know what will happen. During pregnancy something happens, I have to support her. Yeah. I was very happy to support her all the time.” Man M6*

*“I think was kind of sentimental in a way she enjoys it. I mean, when I attended with her, you know, I have to support her, but mostly sentimental.” Man M2*

They also performed a functional role through driving to the appointments, navigating Australian health services and helping to interpret during healthcare consultations.

*“The only thing that you know, that I experienced was my wife. You know, sometimes tell you on the phone she can't speak properly with limited English” Man M4*

Both men and women also described the male partner’s role as being part of the decision-making team. Shared decision-making about pregnancy, birth and infant health was preferred to one person making the decisions alone.

“*Of course (we) decide together, which is good because everything is shared. The doctor once told me something, first wait I'll ask my husband. Next time I told him like that. I can't decide by myself.” Woman F2*

*“I was there the whole time. From start to finish.. so yeah. They were consulting with me when they performed some decision.” Man M2*

In one instance the man’s decision on maternal healthcare over-ruled the woman’s opinion.

*“That one is easy for me, doctor's advice for us [for the woman to be sterilised]. But my husband refused because of our religion, you cannot stop God what to give to you. That's why maybe I'll use another way [other forms of birth control].” Woman F2*

#### Challenges of including men in perinatal healthcare

Men and women described paid work as a barrier to including male partners in perinatal healthcare. Several men said they would have liked to attend more perinatal health appointments but were not able to because of work commitments.

*“As I was working six days. Most of her visits were in business hours. I wish to go follow her, to chat with me or I to help us. But I couldn’t then because of the job. Sometimes she puts my number on her visits and my email and I receive some correspondences from the hospital.” Man M3*

Many participants described the amount of paternity leave they had access to as brief in comparison to the needs of their family at that time. Most were able to take advantage of the government’s 10 days’ paid leave, but two did not meet the eligibility criteria. This particularly constrained their ability to attend the MCH appointments.

*“But here is two weeks for paternity leave. Here is two weeks. Yeah. It's kind of you can only do so much.” Man M2*

It was more likely men would take time off to attend antenatal check-ups if their partner was unwell or there were concerns or complications with the pregnancy.

Both women and men said that after the baby was born it was more of a priority to work and earn money than sacrifice that time to attend the appointments, but if they were available they would attend.

*“If you don't have work, of course you come in, but we don't let him stop work and come with me.” Woman F6*

Men described the responsibility of being the breadwinner and also attempting to take care of their partners and children physically and emotionally through the perinatal period, as challenging and stressful.

*“Because here in Australia, I have to look after my kids and my wife. And I go to work to get income to support my family. So a lot of stress.” Man M6*

The other barrier described was related to cultural norms. This was most often described in the context of other families (not the participants). It was described around some father’s reluctance to engage with young babies, because they were not comfortable, or some families still living in traditional ways.

*“Some African men, they don't have it culturally. I think culturally. Yeah. They're not interested in involving the maternal.. Yeah even here. There are some men they don't hold a newborn baby until two 3 months. Oh yeah. Yes. Only two months. They're very afraid. I don't know. They just feel like it should be with the mom.” Woman F7*

#### Attitudes towards male partner involvement in the perinatal period and healthcare

From almost all participants there was a willingness and positive attitude towards including male partners in perinatal healthcare. This was described as an important action for men to take, with examples and reasons why they should be included.

Participants said there was a lot of work involved in pregnancy, childbirth and raising children and without support, this would be too much for one person.

*“you are responsible for everything from pregnancy and childbirth and then raising children. So it would be very, very, very difficult for a single person” Man M4*

They also said it was important to be involved to form a strong bond between the couple.

*“I have been there together with her until it's finished. So I think it is good for every father to see that and how a woman's suffering during birth. It's very nice to watch and how they give birth. It's very, very tough moment for them….. To bring good family, to grow up with a good family it needs bond. A good bond with a wife to look after each other. You know what I mean? Do you understand?” Man M6*

Commonly, participants indicated that men should be involved to help them understand the suffering and challenge involved in pregnancy, birth and caring for children.

*“And also here, we understand what mothers mean. It’s a tough job for women, a tough job. Yeah ... until you get the baby, you don't know how the mother feels.” Man M2*

Women said engaging men in perinatal healthcare was an opportunity to educate men with the same information they received when attending appointments. This was related to information particular to their individual case, but also general information regarding risks, parenting techniques and services.

*“They have to involved. Because the nurse, she tells you everything about the kids. They're growing everything. They give you information. Yeah. So the father also they have to know that one. Otherwise, you know, one person is not good for everything. It's good for the child because sometimes we left with him. We go outside. So you have to know about the kids growing up, you know? Yeah. You have to involve together.” Woman F1*

Participants also said it was important for men to be involved in the perinatal period overall, to support fathers’ bonding and relationship with their children.

*“And in terms of like even like bonding with the kids, if you like to in everything you like changing nappies and the like, like giving them the shower to kids, like everything you have. If you participate in everything, then I think you feel that you oh, you put in everything. It's a lot better. So it's for you, for your partner, for the baby. It would be very good to involve fathers in every aspect.” Man M4*

However, although all the participants were supportive of male partner involvement, could give examples of how they or their partners were positively involved and could explain how it was beneficial, several also mentioned that it was not always this way in the Ethiopian community. At least one man and several women said that not all Ethiopian men were actively engaged in the perinatal period or in domestic tasks involved with raising children.

*“I see in this country, there are some people living traditionally, like in some African countries ..*.” *Man M2*

One participant said that now in Australia, women worked outside the home, and they were still responsible for doing all the child-care and housework. She suggested that some men were not willing to change and when these issues were raised the couple risked arguments. She felt that women often ended up taking on unfair shares of responsibility to ‘keep the peace’ and avoid conflict.

*“When they speak out. It doesn't go very well. Right. Maybe their partners are resistant to it. They don't really want it. They really don't want to know that, they don't accept it. So it's going to bring arguments.” Woman F7*

One woman’s suggestion to address what she perceived as inequality was to provide education and information directed to men.

*“…Yeah, but especially for African community. I want them. If there is a special short course like in. I can't express it at present. I want the men to get involved and help the wives. Yes. Because having children more than two. It's not easy. Like if they can if they can attend, 3-4 session. How hard is for a man to handle everything?” Woman F7*

## Discussion

There is currently a gap in the research literature regarding father involvement in culturally diverse communities in high income settings. This study contributes evidence about Ethiopian fathers’ experiences of perinatal healthcare in Australia. It explores the attitudes about involving men in pregnancy, childbirth and infant healthcare among parents of young children in the Ethiopian community. The qualitative approach allowed themes to emerge from participants’ experiences, which is essential when research is at an emergent stage and understanding of a minority cultural groups perspective is sought [51].

Key themes included: The differences in fathers involvement between antenatal care (ANC), childbirth and MCH appointments. Participants provided examples of cultural sensitivity, as well as racial stereotyping among healthcare staff. The key barrier to engaging in perinatal healthcare among men was paid employment, lack of flexible working conditions and insufficient paternity leave. Overall there was support from men and women about including men in perinatal healthcare, and the perinatal period more generally. Participants talked about the loss of extended family through migration, new roles for both parents and the need to establish ‘family-like’ relationships with friendship groups in Australia.

Similar to findings generated with women from CALD backgrounds [28, 33, 40, 43], and research in low and middle income settings [26] in this group, men performed roles during perinatal healthcare that included emotional support, logistic support (language translation or driving) and being a part of the shared decision making process. In contrast to findings from low and middle income settings, there was a greater expectation that men would be involved in perinatal healthcare in Australia.

There were notable differences in the findings compared to literature reporting on experiences of men from the *majority* cultural background in high income settings. In the perinatal healthcare setting in Sweden [13], Australia [12] and the UK [52] men reported feeling marginalised, like the care was not father-inclusive, and processes and systems did not allow emotional and physical space for their presence as a partner and parent [12, 13, 15, 52]. The participants in the current study did not report feeling marginalised, instead they most often reported positive experiences where they were satisfied with the care shown to them during antenatal care, childbirth and postnatal care. A possible explanation for the differences reported is the level of expectations that the Ethiopian partners had in relation to their involvement in perinatal healthcare. Many had knowledge or experience of perinatal healthcare overseas and reflected favourably on many aspects of healthcare in Australia. It is likely that social norms of father’s involvement in families are more established in locations such as Sweden, Australia and the United Kingdom when compared to Ethiopia. Men and women in the Ethiopian-Australian community had modest expectations about how men should be included during pregnancy, childbirth and infancy, and their expectations were met.

Descriptions of social networks in Ethiopia and Australia, elucidated in this research, reflect different models of family structure in the respective cultures [53, 54]. In Australia the primary family structure is the nuclear family. Migration without extended family members, places additional demands on mothers and fathers. Participants in this research tended to adapt their functioning to take on a more nuclear family model with additional roles for the parents, describing the male partner as the key support person. This is consistent with reports from studies conducted with women from a CALD background, where research indicated fathers’ role in relation to pregnancy, childbirth and the postnatal period had changed in comparison to in their home country [28, 33] and that women described their male partners as the main support person in Australia because they lacked support from an extended network they would have had in their home country [27, 28].

In addition to new roles for the parents, there were consistent accounts of participants also re-created an extended family network through close friends they described as family. This is also consistent with literature documenting migrant experiences in Australia. A recent qualitative study of 164 migrants from seven collectivist cultural communities concluded that extended community networks mitigated culture shock and a social capital framework was appropriate to understand the experiences of these communities [55].

These findings suggest that families migrating from collectivist cultures may benefit from support for changing roles of men and women in relation to pregnancy, childbirth and infancy inside the family unit, as well as support in cultivating an extended support network in their cultural group. Although fathers did feel welcomed, these fathers would benefit from inclusive perinatal education and emotional support from health services. Backing from the wider community and institutions to cultivate broader social networks within their own cultural communities is also important. Supporting families to re-build an extended family network in a new country allows them to flourish in a way that is culturally sensitive. This can be achieved through government support for community led events and organisations, such as sporting events, religious institutions, language schools, language-specific playgroups and cultural celebrations. Support of these social networks and new roles within the family helps prevent mental health concerns in the perinatal period.

There were mixed experiences of culturally competent care delivered by healthcare staff. With examples of cultural sensitivity, as well as racial stereotyping. Two participants were able to perceive differences between healthcare professionals, reporting midwives were more caring than specialists. These findings suggest that although Australian perinatal healthcare has progressed over the preceding decades to be more culturally sensitive and inclusive, there is still a need for cultural competency training and education for practicing professionals and those training to enter the workforce. This training should include specialists and those working in private practice. The need for improvements in cultural competency is consistent with reviews in the area, suggesting families of CALD background are more likely to encounter negative experiences [24].

Part of delivering culturally competent care requires practitioners to respect the cultural differences in matters such as decision making. This can be a difficult area to navigate when part of the perinatal period pertains to women’s health and bodies. A non-judgemental respect of cultural differences while supporting a women’s right to autonomy, is necessary in supporting men’s involvement in maternal healthcare.

Similar to previous research with women in CALD communities, where women mentioned that their partners needed to work long hours and were thus unable to provide enough support to ‘fill the gap’ left by absent extended family [27, 35, 40], or where they considered men to be the provider more than the nurturer for the family [34, 41]. The key barrier in this study to engaging in perinatal healthcare among men was paid employment, lack of flexible working conditions and insufficient paternity leave. These barriers could be overcome through industry policies recognising the role of fathers (e.g. carers leave, support for flexible working conditions and company paid paternity leave). They could also be navigated by flexible delivery of MCH appointments. Appointments held outside of business hours or using other delivery methods such as including the fathers on the phone or online platforms could allow more fathers to be involved.

There were differences in descriptions of ANC, childbirth and MCH appointments, suggesting that different approaches are needed to support men’s involvement in each type of healthcare. Participants reported mixed experiences of ANC. Men reported overall positive experiences of healthcare during childbirth and felt sufficiently included. The interviews revealed more barriers to attendance during MCH appointments, although this was not always framed negatively. The findings suggest that strategies to include men in perinatal healthcare could be prioritised towards MCH appointments, as these had the least engagement, or alternative sources of information could be targeted towards men in this time to compensate for missing the MCH appointments. Consulting with men about their preferences is important. In general, fathers find it difficult to identify which resources are reputable, which advice to follow [56].

There was support from men and women about including men in perinatal healthcare, and the perinatal period more generally. Participants suggested that including men supported the couple’s relationship and the father’s bond with their child, allowed men to understand the processes involved with pregnancy, childbirth and infant-care and improve their health literacy. This demonstrates a willingness to involve male partners in perinatal healthcare among men and women in the Ethiopian community and knowledge of the benefits. This should encourage services to maintain and increase strategies to engage fathers from this group.

However, consistent with findings from Sub-Sahara Africa [26], some participants explained that cultural attitudes may determine that pregnancy, childbirth and infancy are regarded as a woman’s domain in the Ethiopian community. This could be a barrier to men’s involvement in perinatal healthcare in some cases. A culturally competent approach would suggest that healthcare workers should be sensitive to this attitude and allow families to determine the roles performed by members of the family in the perinatal period.

### Limitations

As a qualitative study, the findings are reflective of the group’s accounts, but they are not generalizable to the population. The current study supports the design of quantitative research with a larger sample. A limitation is the recruitment strategy (snowball) which did not encourage participation from people isolated from the Ethiopian community, suggesting that future projects may compliment this approach with alternative recruitments strategies targeting more isolated families. Previous research identified that significant proportions of women from a CALD background in certain communities were divorced or widowed and did not have a male partner [36, 44, 45], suggesting that studies of male involvement may not reflect their experiences.

## Conclusion

In conclusion, Ethiopian families living in Australia flourish during pregnancy, childbirth and infancy through positive father involvement, and by creating an extended support network among Ethiopian friends. There is a willingness to involve male partners in the Ethiopian community in Australia, although it is recognised as a cultural change. Experiences of male partner involvement are mixed among healthcare types, with men attending MCH appointments less frequently than antenatal appointments. This suggests men may be missing out on the education provided during these appointments and may benefit from an alternative. There are not universally high levels of cultural competency among healthcare professionals, with further training still required. Commitment to paid employment remains a barrier to men’s involvement, suggesting that flexible working conditions and increased paternity leave would support their involvement. Alternatively services could utilise flexible delivery methods such as phone and zoom to include fathers.

## Data Availability

The datasets generated and/or analysed during the current study are not publicly available due confidentiality but are available from the corresponding author on reasonable request.

## Abbreviations

CALD: Culturally and Linguistically Diverse
MCH: Maternal and Child Health
ANC: Antenatal care
